# COVID-19 Vaccine Perception in South Korea: Web Crawling Approach

**DOI:** 10.2196/31409

**Published:** 2021-09-08

**Authors:** Hocheol Lee, Eun Bi Noh, Sung Jong Park, Hae Kweun Nam, Tae Ho Lee, Ga Ram Lee, Eun Woo Nam

**Affiliations:** 1 Yonsei Global Health Center, Yonsei University Wonju Republic of Korea; 2 Department of Health Administration, Yonsei University Graduate School Wonju Republic of Korea; 3 Department of Information Statistics, Yonsei University Graduate School Wonju Republic of Korea; 4 Department of Preventive Medicine, Wonju College of Medicine, Yonsei University Wonju Republic of Korea

**Keywords:** COVID-19 vaccine, COVID-19, instagram, social media, infodemiology, sentiment analysis, vaccine perception, South Korea, web crawling, AstraZeneca, Pfizer

## Abstract

**Background:**

The US Centers for Disease Control and Prevention and the World Health Organization emphasized vaccination against COVID-19 because physical distancing proved inadequate to mitigate death, illness, and massive economic loss.

**Objective:**

This study aimed to investigate Korean citizens’ perceptions of vaccines by examining their views on COVID-19 vaccines, their positive and negative perceptions of each vaccine, and ways to enhance policies to increase vaccine acceptance.

**Methods:**

This cross-sectional study analyzed posts on NAVER and Instagram to examine Korean citizens’ perception of COVID-19 vaccines. The keywords searched were “vaccine,” “AstraZeneca,” and “Pfizer.” In total 8100 posts in NAVER and 5291 posts in Instagram were sampled through web crawling. Morphology analysis was performed, overlapping or meaningless words were removed, sentiment analysis was implemented, and 3 public health professionals reviewed the results.

**Results:**

The findings revealed a negative perception of COVID-19 vaccines; of the words crawled, the proportion of negative words for AstraZeneca was 71.0% (476/670) and for Pfizer was 56.3% (498/885). Among words crawled with “vaccine,” “good” ranked first, with a frequency of 13.43% (312/2323). Meanwhile, “side effect” ranked highest, with a frequency of 29.2% (163/559) for “AstraZeneca,” but 0.6% (4/673) for “Pfizer.” With “vaccine,” positive words were more frequently used, whereas with “AstraZeneca” and “Pfizer” negative words were prevalent.

**Conclusions:**

There is a negative perception of AstraZeneca and Pfizer vaccines in Korea, with 1 in 4 people refusing vaccination. To address this, accurate information needs to be shared about vaccines including AstraZeneca, and the experiences of those vaccinated. Furthermore, government communication about risk management is required to increase the AstraZeneca vaccination rate for herd immunity before the vaccine expires.

## Introduction

COVID-19 was first reported in Wuhan in December 2019, and on March 11, 2020, the World Health Organization (WHO) declared it a pandemic. As of May 10, 2021, COVID-19 has spread to 221 countries and 159,145,765 confirmed cases and 3,310,621 deaths have been reported internationally [1]. Furthermore, the global economic loss due to COVID-19 in 2020 was estimated at US $9trillion [2]. Accordingly, the US Centers for Disease Control and Prevention (CDC) and the WHO determined that physical (social) distancing alone was insufficient to prevent and eliminate COVID-19 and stressed the need for vaccination while simultaneously initiating the development of COVID-19 vaccines [3,4].

As of May 17, 2021, 7% of the world’s population have been vaccinated [5]. However, because clinical trials for vaccines advanced quickly, and vaccines were approved in accelerated processes over a short period, negative information regarding COVID-19 vaccines has proliferated [6], due to which the number of people refusing to be vaccinated has increased. Previous studies have examined people’s hesitancy toward vaccines [7-9]. One study [10] reported a variety of significant reasons for vaccine refusal, including lack of trust in the vaccines, deaths due to vaccination, negative rumors about the vaccines, religious beliefs, antigovernment sentiment, public health messaging failure, and a lack of understanding regarding the need for vaccination.

The COVID-19 vaccination rate is rising slowly relative to the initial plans due to incorrect information and negative perception. Thus, there is an opinion that it may have a negative impact on herd immunity in communities [11]. To increase vaccine acceptance, it is necessary to identify the positive and negative aspects of perception regarding COVID-19 vaccination and for governments to respond expeditiously, based on empirical findings. Furthermore, the WHO strongly encourages governments to deliver the accurate information about COVID-19 vaccines to citizens [12]. It is well-known that risk communication using social media, such as Facebook, Twitter, and YouTube, was the most effective way to disseminate information during the SARS epidemic in 2013 [13,14]. That is, governments’ risk communication during the COVID-19 pandemic is critical for increasing the acceptance of nonpharmaceutical approaches and COVID-19 vaccines. Korea is 1 of 5 representative countries that responded successfully to the COVID-19 infection [15]. However, the vaccination rate here is lower, compared with that in other more developed countries, as there was a delay in securing vaccine supplies. Moreover, the vaccine refusal rate is 33%, ranking 64th worldwide. Furthermore, with the extensive coverage of vaccine side effects by the media, negative information has become widespread among citizens [16]. This negative information regarding COVID-19 vaccines is spreading on popular Korean social media platforms—with YouTube being the most common, followed by NAVER and Instagram [17].

In Korea, COVID-19 vaccination commenced on February 26, 2021, initially administered to adults aged over 65 years in long-term care hospitals and nursing homes, and to health care professionals. The country developed the following plan and is currently proceeding as planned: adults aged over 60, pharmacy employees, disabled persons, and homeless persons were vaccinated in Q2; all adults were vaccinated in Q3; and all citizens who were unvaccinated are targeted in Q4 [18].

Since early 2021, 2 types of COVID-19 vaccines, AstraZeneca (AZ) and Pfizer, have been produced in Korea. As of May 10, 2021, 4,181,003 people have been vaccinated—2,014,788 with AZ and 2,166,215 with Pfizer. The vaccine refusal rate in Korea was 33%, and these individuals refused to be vaccinated despite being eligible for COVID-19 vaccination. Hence, Korean vaccine experts predict that it would not be feasible to reach herd immunity against COVID-19 by December 2021, because the proportion of vaccinated persons will not reach 70% [19]. Citizens’ refusal to be vaccinated poses a major problem to the government’s plan.

Accordingly, the purpose of this study was to investigate Korean citizens’ perceptions of COVID-19 vaccines. The specific objectives were to (1) investigate their perception of COVID-19, (2) examine the positive and negative aspects of the perception of each type of vaccine, and finally, (3) provide evidence needed to develop policies to increase vaccine acceptance by examining the current perception of COVID-19 vaccines.

## Methods

### Study Design

**Figure 1:**
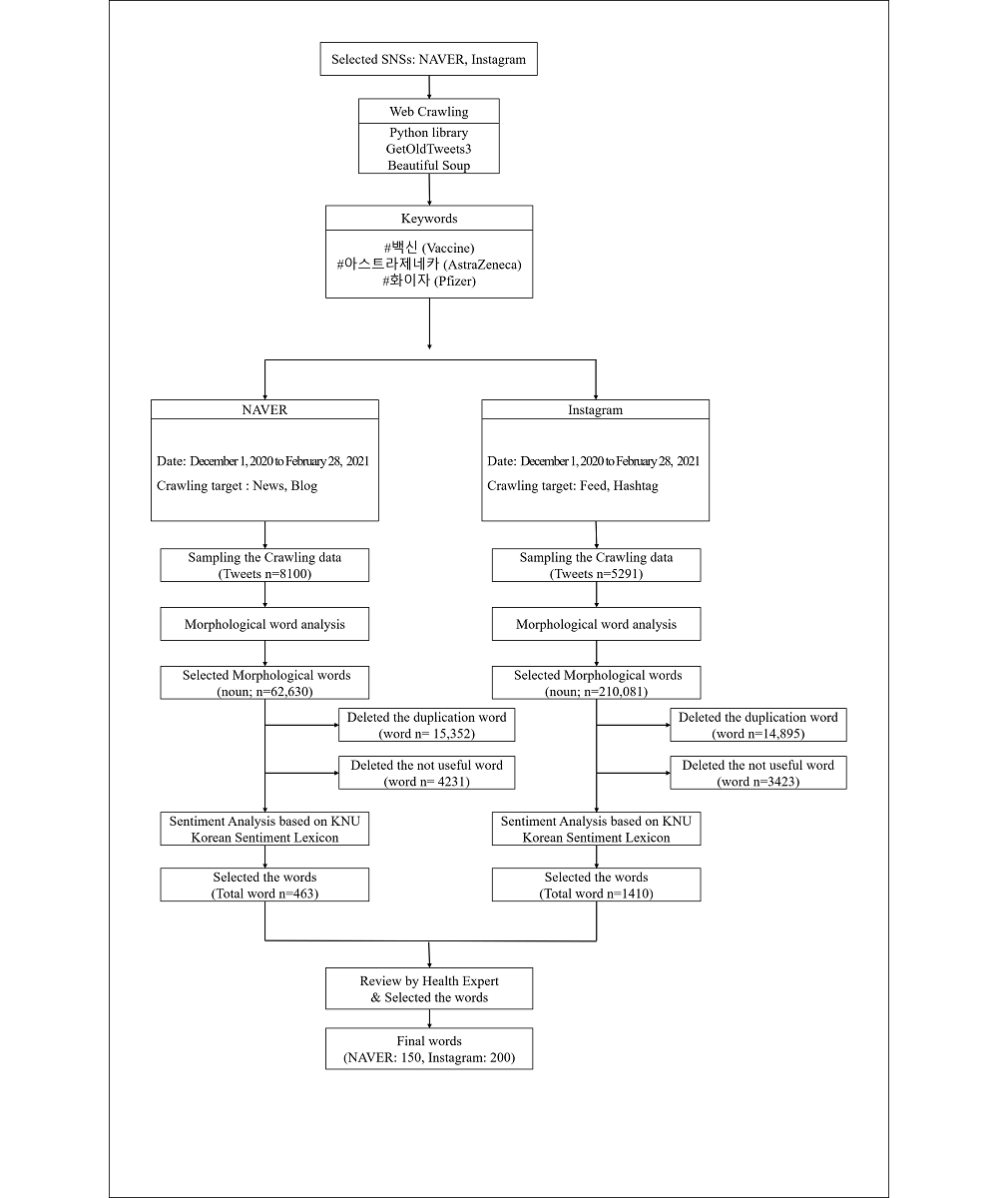
Research flowchart. SNSs: social network sites, KNU: Kunsan National University.

This cross-sectional study analyzed posts uploaded to NAVER and Instagram (2 social network sites [SNSs] available in Korea) between December 1, 2020, and February 28, 2021, to examine Korean citizens’ perception of COVID-19 vaccines. A flowchart of the study is presented in Figure 1.

### Data Collection

To examine the COVID-10 vaccine perception of the participants, who were Korean citizens, their SNS posts were analyzed. Data were collected from the 2 most popular SNSs in Korea: NAVER and Instagram. Posts uploaded to NAVER blogs and news and Instagram feeds between December 1, 2020, and February 28, 2021, were collected. To compile the data, web crawling was performed using Requests in Python 3.8.3 Library, Beautiful Soup, and Webdriver. The keywords utilized were “vaccine,” “AstraZeneca,” and “Pfizer.” The search was performed using the search bar in NAVER and the hashtag search in Instagram.

A total of 8100 posts in NAVER and 5291 in Instagram were sampled through web crawling. Morphology analysis was performed, and the NAVER posts were classified into 62,630 words and Instagram posts into 210,081 words. Overlapping or meaningless words were removed, resulting in 463 words from NAVER and 1410 words from Instagram. Then, sentiment analysis was performed, and 3 public health professionals reviewed the results. Finally, 150 words from NAVER and 200 words from Instagram were included in the analysis.

### Statistical Analysis

The words were collected from 2 representative SNSs in Korea, NAVER and Instagram, and were categorized as positive or negative for the purpose of analysis. To classify the words as positive or negative, text mining was performed based on the KNU Korean Sentiment Lexicon [20].

The KNU Korean Sentiment Lexicon, created by the Kunsan University in Korea, is an emotional dictionary consisting of positive and negative words that are used to express people’s basic emotions. Each word in the emotional dictionary was determined through the consensus of evaluators using a Likert 5-point scale—“very negative,” “negative,” “neutral,” “positive,” and “very positive”—ranging from 2 (very positive) to –2 (very negative). Based on the score, each emotional expression is classified as either positive or negative.

Next, the rankings of the words classified as positive or negative were visualized separately for “vaccine,” “AstraZeneca,” and “Pfizer,” using the word cloud technique. Positive and negative words that were used with the keywords were ranked based on their frequency.

Lastly, the words that were common to “AstraZeneca” and “Pfizer” were visualized by presenting the words associated with AZ on the x-axis and those associated with Pfizer on the y-axis to show word frequency according to the type of vaccine.

## Results

### Crawling Data Characteristics

In this study, to investigate vaccine acceptance, web crawling was performed using the keywords “vaccine,” “AstraZeneca,” and “Pfizer” on posts in 2 SNSs available in Korea (Instagram and NAVER) between December 1, 2020, and February 28, 2021. A total of 5291 Instagram posts and 8100 NAVER posts were sampled (Table 1).

The 7-day period during which the largest volume of data was collected from Instagram, 998/5291 posts (18.86%), was between February 22, 2021 and February 28, 2021. From NAVER, the data were collected uniformly for approximately 630/8100 (7.78%) posts per period.

**Table 1:** The frequency of crawling data.

Date	Instagram (n=5291)	NAVER (n=8100)
	Crawling data, n (%)	Crawling data, n (%)
December 1-7, 2020	239 (4.52)	630 (7.78)
December 8-15, 2020	496 (9.37)	630 (7.78)
December 16-21, 2020	447 (8.45)	630 (7.78)
December 22-28, 2020	379 (7.16)	630 (7.78)
December 29-31, 2020	216 (4.08)	270 (3.33)
January 1-7, 2021	300 (5.67)	630 (7.78)
January 8-15, 2021	355 (6.71)	630 (7.78)
January 16-21, 2021	429 (8.11)	630 (7.78)
January 22-28, 2021	282 (5.33)	630 (7.78)
January 29-31, 2021	187 (3.53)	270 (3.33)
February 1-7, 2021	287 (5.42)	630 (7.78)
February 8-15, 2021	253 (4.78)	630 (7.78)
February 16-21, 2021	423 (7.99)	630 (7.78)
February 22-28, 2021	998 (18.86)	630 (7.78)

### Crawling Data Ranking

Of the words collected separately by using the keywords “vaccine,” “AstraZeneca,” and “Pfizer,” the 20 most frequent words are summarized in Table 2. The 20 most frequent words that were crawled with “vaccine” appeared 2323 times. The frequency of the top 20 words crawled with “AstraZeneca” and “Pfizer” were 559 and 486, respectively.

**Table 2:** Ranking of the crawled data according to word frequency for each vaccine type.

Rank	Type						Vaccine (n=2323)	
	AstraZeneca (n=559)			Pfizer (n=486)				
	Word	n (%)	Word		n (%)	Word		n (%)
1	Safe effect	163 (29.2)	Escape		179 (36.8)	Good		312 (13.4)
2	Possibility	47 (8.4)	Difficult		39 (8.0)	Treatment		231 (9.9)
3	Safety	45 (8.1)	Achieved		39 (8.0)	Health		217 (9.3)
4	Prevention	42 (7.5)	Good		35 (7.2)	Safety		215 (9.3)
5	Treatment	26 (4.7)	Abnormal		26 (5.3)	Death		145 (6.2)
6	Trust	23 (4.1)	Pain		19 (3.9)	Prevention		139 (5.9)
7	Anxiety	22 (3.9)	Peace		18 (3.7)	Possibility		137 (5.9)
8	Difficult	22 (3.9)	No		16 (3.3)	Safe effect		123 (5.2)
9	Refusal	20 (3.6)	Giving up		15 (3.1)	Tough		103 (4.4)
10	Distrust	20 (3.6)	Having a cold		11 (2.3)	Risk		90 (3.9)
11	Ill	19 (3.4)	Value		11 (2.3)	Infected		90 (3.9)
12	Health	17 (3.0)	Fainting		10 (2.1)	Recovery		80 (3.4)
13	Increase	15(2.7)	Need		10 (2.1)	Rise		73 (3.1)
14	Concerned	13 (2.3)	Risk		9 (1.9)	Happy		62 (2.7)
15	Stability	12 (2.1)	Limit		9 (1.9)	Hope		56 (2.4)
16	Shortage	11 (2.0)	Convulsion		8 (1.6)	Overcoming		55 (2.4)
17	Okay	11 (2.0)	Righteous Person		8 (1.6)	Late		55 (2.4)
18	Experts	11 (2.0)	Cautious		8 (1.6)	Anxiety		49 (2.1)
19	Overcoming	10 (1.8)	Improvement		8 (1.6)	Illness		46 (2.0)
20	Recovery	10 (1.8)	Understanding		8 (1.6)	Banned		45 (1.9)

Among the words crawled with “vaccine,” “good” ranked first, with a frequency of 312/2323 (13.43%). The words that ranked second to fifth were “treatment” (231/2323, 9.94%), “health” (217/2323, 9.34%), “safety” (215/2323, 9.26%), and “death” (145/2323, 6.24%), respectively.

Of the words crawled with “AstraZeneca,” “side effect” ranked first, with a frequency of 163/559 (29.2%), followed by “possibility” (47/559, 8.4%), “safety” (45/559, 8.4%), “prevention” (42/559, 7.5%), and “treatment” (26/559, 4.7%).

Of the words crawled with “Pfizer,” “escape” was the most frequent (179/486, 36.8%). The words ranked second to fifth were “difficult” (39/486, 8.0%), “achieved” (39/486, 8.0%), “good” (35/486, 7.2%), and “abnormal” (26/486, 5.3%), respectively.

### Classification of Crawled Data Into Positive and Negative Words

The crawled data were classified as positive or negative using a positive/negative classification system and by consulting with 3 public health experts (Table 3).

**Table 3:** Counts and frequencies of positive and negative words in the crawled data.

	Type							Vaccine			
	Pfizer			AstraZeneca						
	Word count (n=122)	Frequency (n=885)	Word count (n=89)		Frequency (n=670)	Word count (n=146)			Frequency (n=3698)		
Positive	47 (38.5)	387 (43.7)	37 (41.6)		194 (29.0)	43 (29.5)			1981 (53.6)		
Negative	75 (61.5)	498 (56.3)	52 (58.4)		476 (71.0)	103 (70.5)			1717 (46.4)		

Of the words crawled with “vaccine,” 103/146 (70.5%) were classified as negative and 43/146 (29.5%) as positive. Thus, there were more negative words. However, positive words were used more frequently (1981/3698, 53.57%).

Of the words crawled with “Pfizer,” 75/122 (61.5%) were classified as negative and 47/122 (38.5%) as positive; thus, there were more negative words in the data. Negative words were used more frequently (498/885, 56.3%).

Of the words crawled with “AstraZeneca,” 52/89 (58%) were classified as negative and 37/89 (42%) as positive; thus, there were more negative words. Again, negative words were more frequently used (476/670, 71.0%) than positive words (194/670, 29.0%).

With respect to “vaccine,” positive words were more frequently used than negative words; however, regarding “AstraZeneca” and “Pfizer” negative words were more frequently used than positive ones.

**Figure 2:**
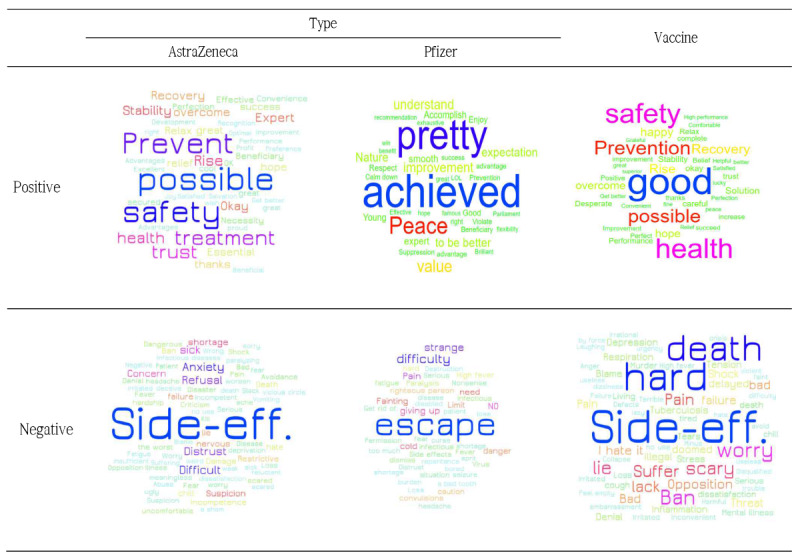
Word cloud visualizations of crawled data. Side-eff: side-effects.

Word cloud visualizations (Figure 2) were created separately for positive and negative words classified based on the crawled data with the keywords of “vaccine,” “AstraZeneca,” and “Pfizer.” Regarding “vaccine,” positive words were “good,” “safety,” “hope,” “recovery,” and “overcoming,” and negative words were “side effect,” “tough,” “death,” “concerned,” and “lies.”

For AZ, positive words included “possibility,” “safety,” “prevention,” “treatment,” and “trust,” and negative words were “side effect,” “anxiety,” “difficult,” and “refusal.” With respect to Pfizer, positive words were “achieved,” “good,” and “value,” and negative words were “escape,” “difficult,” “pain,” and “giving up.”

**Figure 3:**
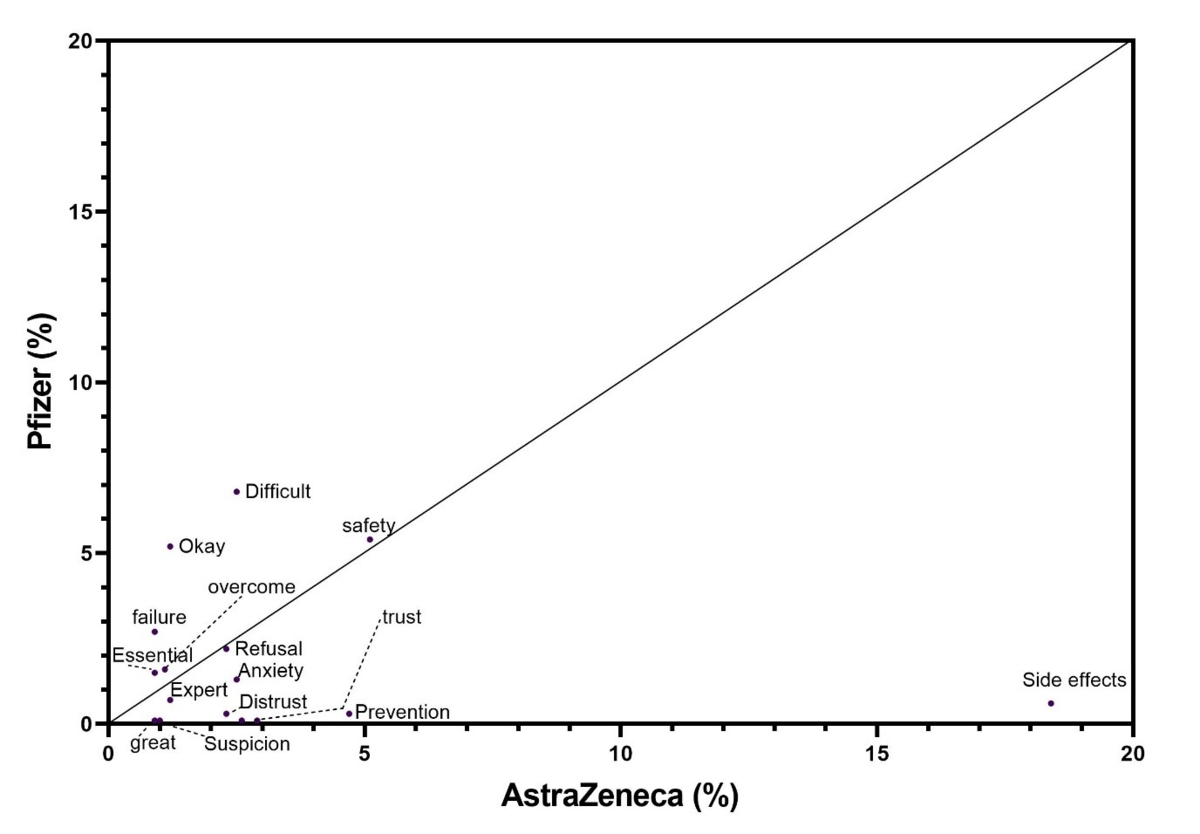
Comparison of the crawled words for the AstraZeneca and Pfizer vaccines.

Of the positive and negative words crawled with the vaccine types, “AstraZeneca” and “Pfizer,” as keywords, those found for both types of vaccine were examined for their frequencies (Figure 3). A total of 16 words were commonly associated with AZ and Pfizer. Of those, “side effect” showed the highest frequency (163/559, 29.2%) for AZ. By contrast, the frequency of “side effect” for Pfizer was 0.6% (4/673). Additionally, “prevention,” “treatment,” “trust,” “anxiety,” and “distrust” demonstrated higher frequencies for AZ compared with Pfizer.

However, “difficult,” “okay,” “failure,” “safety,” “overcoming,” and “essential” were more frequently used with Pfizer compared with AZ.

## Discussion

### Principal Findings

The purpose of this study was to (1) examine Korean citizens’ perceptions of COVID-19 vaccines, (2) identify their overall views of the vaccines including the positive and negative aspects of their perceptions, and (3) provide evidence for policy development to increase COVID-19 vaccine acceptance.

To do so, a web crawling approach was used to collect data from NAVER and Instagram using “vaccine,” “AstraZeneca,” and “Pfizer” as the keywords. In a previous study using the existing web crawling technique to analyze citizens’ perceptions, data were collected from a variety of SNSs, including Google Trends, Twitter, and Facebook [21]. However, our study crawled data from the most popular SNSs in Korea: NAVER and Instagram.

For the data crawled with “vaccine,” the proportion of positive words (1981/3698, 53.57%) was higher than that of negative words (1717/3698, 46.43%), which revealed that citizens’ perceptions of vaccination is somewhat positive. According to a study that examined public perception in Bangladesh based on over 10,000 Facebook posts using “vaccine” as the keyword [22], the proportion of citizens who regarded vaccination positively (74.61%) was similar to this study’s findings. Of the positive words used in the posts, “nice” was most regularly used (13.4%), followed by “treatment” (9.9%), “health” (9.3%), “safety” (9.3%), “prevention” (6.0%), “recovery” (3.4%), and “hope” (2.4%). The findings showed positive expectations regarding prevention, elimination, and treatment through vaccination against COVID-19.

By contrast, the results of the analysis conducted in this study with the 2 vaccine types available in Korea, AZ and Pfizer, showed that negative perception was stronger, as shown by the frequency of negative words associated with AZ and Pfizer: 71.0% (476/670) and 56.3% (498/885), respectively. This finding is consistent with that of a previous study—that is, negative viewpoints were more prevalent in Korean citizens and that there was a stronger negative perception regarding the AZ vaccine [23]. The public’s perception became negative due to reports of people developing thrombocytopenia after receiving the AZ vaccination. In particular, the perception changed negatively in people who were still deciding whether to be vaccinated [24]. Additionally, this study found that Korean citizens were concerned about the side effects of AZ, and therefore tended to refuse it, as revealed by the finding that words widely associated with AZ included “side effects,” “anxiety,” and “refusal.”

As of May 2021, Korea secured AZ and Pfizer vaccine supplies and initiated vaccinating health care professionals and people aged 60 years or older. By May 20, 2021, 2% of the general population were vaccinated [25]. The Korean government is planning to vaccinate at least 70% of the population by December 2021 to achieve herd immunity.

Several studies have emphasized the need for mass acceptance of vaccination to achieve the goal of herd immunity [26]. However, as shown in this study, there is an intense negative perception about AZ and Pfizer vaccines in Korea. Research indicates that the main cause of such a negative viewpoint is the failure of the government to communicate risk [27].

Risk communication is a component of a country’s preparedness, proposed by the WHO, for infection prevention, control, and management [28]. The Middle East respiratory syndrome coronavirus (MERS-CoV) outbreak in Korea is a representative of the impact of national capacity for risk communication during an outbreak. During the MERS-CoV outbreak, the Korean government promptly shared information with citizens, and citizens’ trust in the information played a crucial role in preventing the spread of the infection [29]. Since MERS-CoV, in 2017, Korea received a score of 3.6 out of 5 points by the Joint External Evaluation, a WHO evaluation system for risk communication [30]. During the current COVID-19 pandemic, Korea demonstrated excellent risk communication capacity based on the experience with MERS-CoV and was named, along with New Zealand, Australia, and Taiwan, as a country that successfully responded to the COVID-19 pandemic [31]. However, regarding the COVID-19 vaccination policy, the psychology of refusal is widespread, with 1 of 4 people refusing to be vaccinated. According to an online survey conducted with 1093 Korean adults [32], 62.6% of the respondents trusted the government’s effort for vaccination. This level was similar to our study’s finding regarding trust (1981/3698, 53.57%) based on data crawling with the keyword “vaccine.” Furthermore, 70.5% of respondents in the study indicated that the Pfizer vaccine was safe, while 30.4% responded that the AZ vaccine was safe [32]. This finding is consistent with the findings of this study regarding a negative perception of AZ (476/670, 71.0%). Moreover, in the online survey, side effects were the primary reason for the negative perception of AZ, which concurs with the findings of this study. According to the studies conducted by the manufacturers/developers of AZ, only 28 out of 17 million people vaccinated with AZ experienced side effects; therefore, side effects are not a serious concern. The WHO, US CDC, and Korea Disease Control and Prevention Agency (formerly Korea Centers for Disease Control and Prevention [KCDC]) strongly recommend AZ [33,34]. However, trust in the government’s risk communication decreased, and the vaccination program slowed down. In the United States, the “lack of trust in information delivered by the government” was the second most common (12.5%) reason for citizens’ reluctance toward getting vaccinated against COVID-19 [35].

Thus, this study makes the following 3 suggestions to increase COVID-19 vaccine acceptance and to achieve herd immunity. The first is to share the cases vaccinated with the AZ in anticipation of a bandwagon effect. The stakeholders who make decisions regarding COVID-19 vaccination policy (including the president, high-ranking officials) can promote safety after being vaccinated with AZ. It has been reported that celebrities and entertainers sharing their experiences in infomercials are also effective [36].

Second, risk communication is a valuable tool to promote policies and increase trust in the government. The government should not only accurately and rapidly provide information regarding COVID-19 vaccines, but should also share evidence-based, reliable information to increase citizens’ trust. Additionally, when promoting the COVID-19 vaccination policy, the gap between experts and non-experts in terms of risk information should be considered, and messaging should be strategically presented to aid in understanding the risks.

Finally, it is suggested that incentives be provided to persons who are vaccinated. Korea signed a priority contract with AZ to secure vaccine supplies. Because AZ has a short shelf life, vaccines that have passed the expiry date should be discarded if vaccination does not progress as planned. Fortunately, smartphone penetration is high in Korea, and if the person to be vaccinated misses their appointment, the next person in the vaccine registration list is notified through a smartphone notification. In Korea, this is termed “No Show.” Providing incentives for people who are vaccinated ought to be considered to increase AZ acceptance within a specified time, and to change people’s perceptions.

This study has a few limitations. First, the data were obtained from NAVER and Instagram; thus, there is a limitation in representativeness. Because internet users tend to be young, the opinions of older people were not fully reflected in the study’s findings. Second, only the texts posted on the internet were analyzed, and the study’s findings do not reflect various demographic characteristics, educational levels, and access to health information of the people who posted the texts. In future research, nationwide survey studies should be performed by considering these limitations and factoring in characteristics of the study’s participants. Third, because the “KNU Korean Sentiment Lexicon” is a latest word classification tool in Korea, the number of studies pertaining to COVID-19 that have used this tool is limited. Hence, more studies are needed on the words that are classified as either positive or negative in this tool.

### Conclusion

This study examined COVID-19 vaccine acceptance in Korea using a web crawling approach with 3 keywords: “vaccine,” “AstraZeneca,” and “Pfizer.” It was found that 71.0% (476/670) of the words crawled with “AstraZeneca” were classified as negative, and the proportion of negative words associated with Pfizer was 56.3% (498/885). Side effects were found to be the greatest concern regarding AZ. To address this problem, accurate information sharing about COVID-19 vaccines, including AZ, is suggested. Additionally, it is suggested that the experiences of people who are vaccinated should be shared in anticipation of a bandwagon effect. Finally, the government ought to increase the AZ vaccination rate by managing communication about risks so that vaccination occurs before the vaccine expires.

## Abbreviations

AZ: AstraZeneca
CDC: US Centers for Disease Control and Prevention
KCDC: Korea Centers for Disease Control and Prevention
MERS-CoV: The Middle East respiratory syndrome coronavirus
SNSs: social network sites
WHO: World Health Organization
